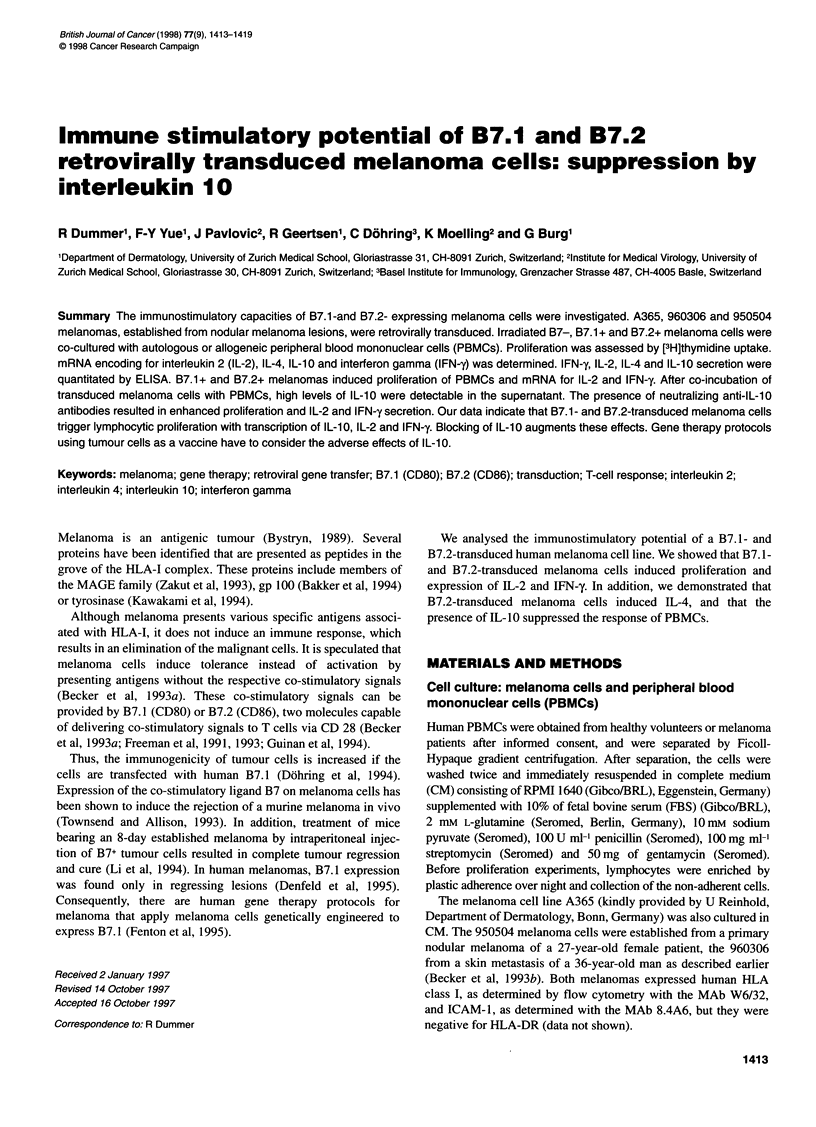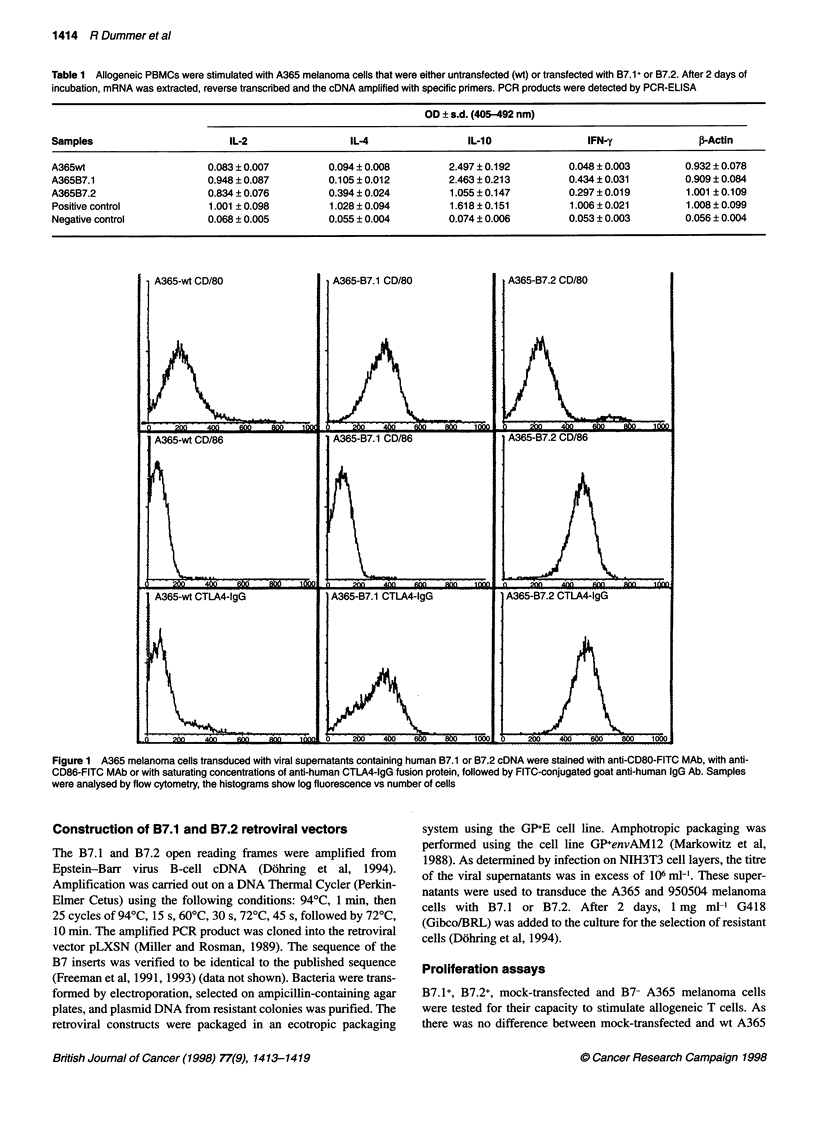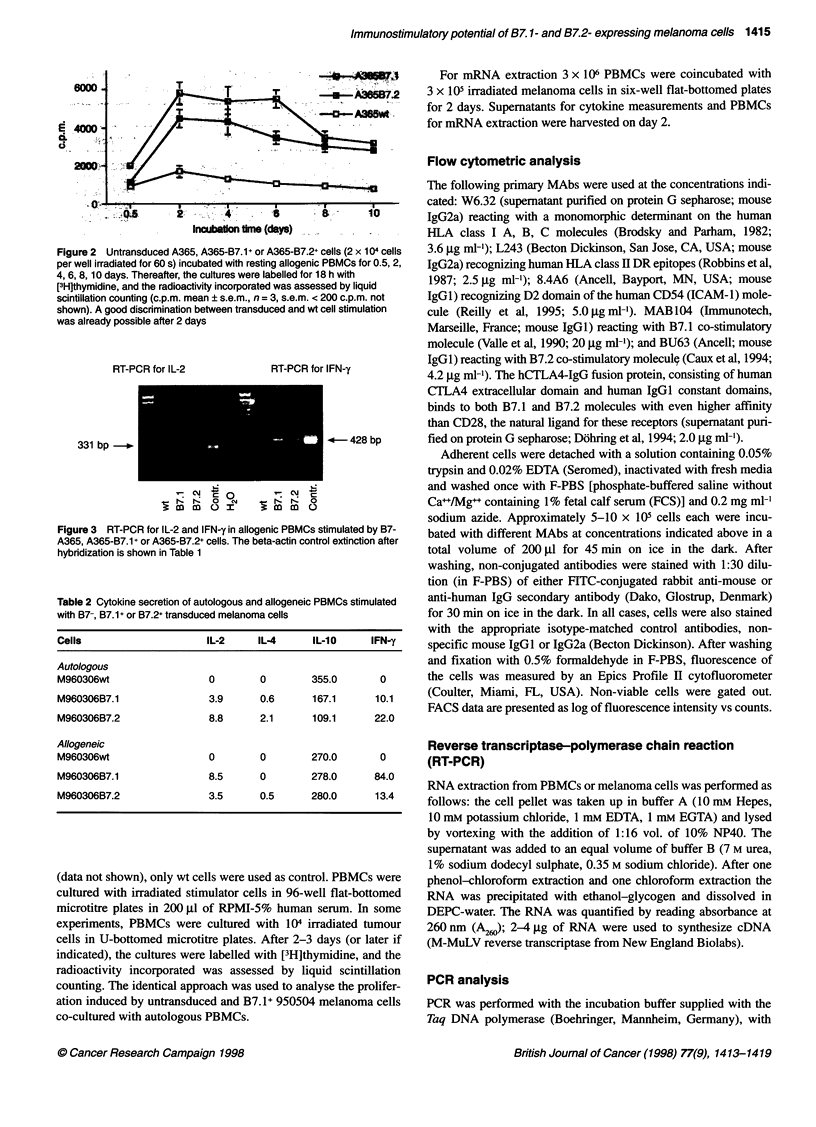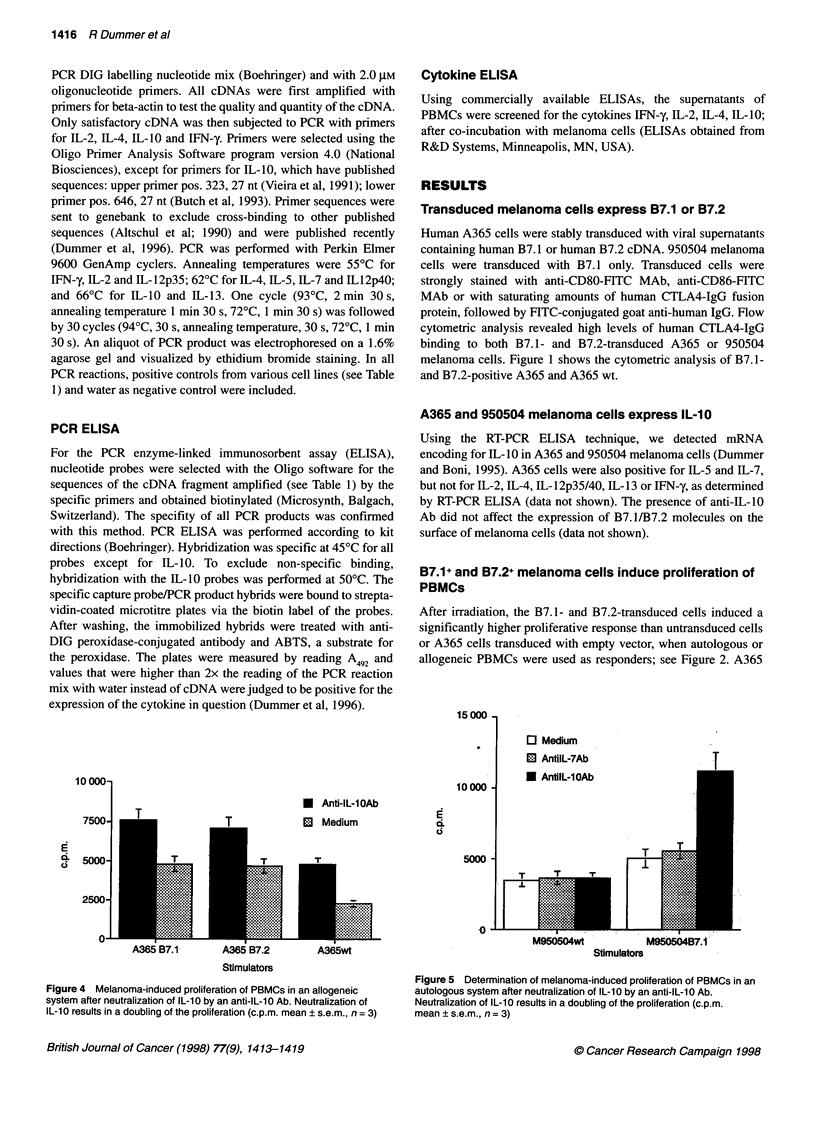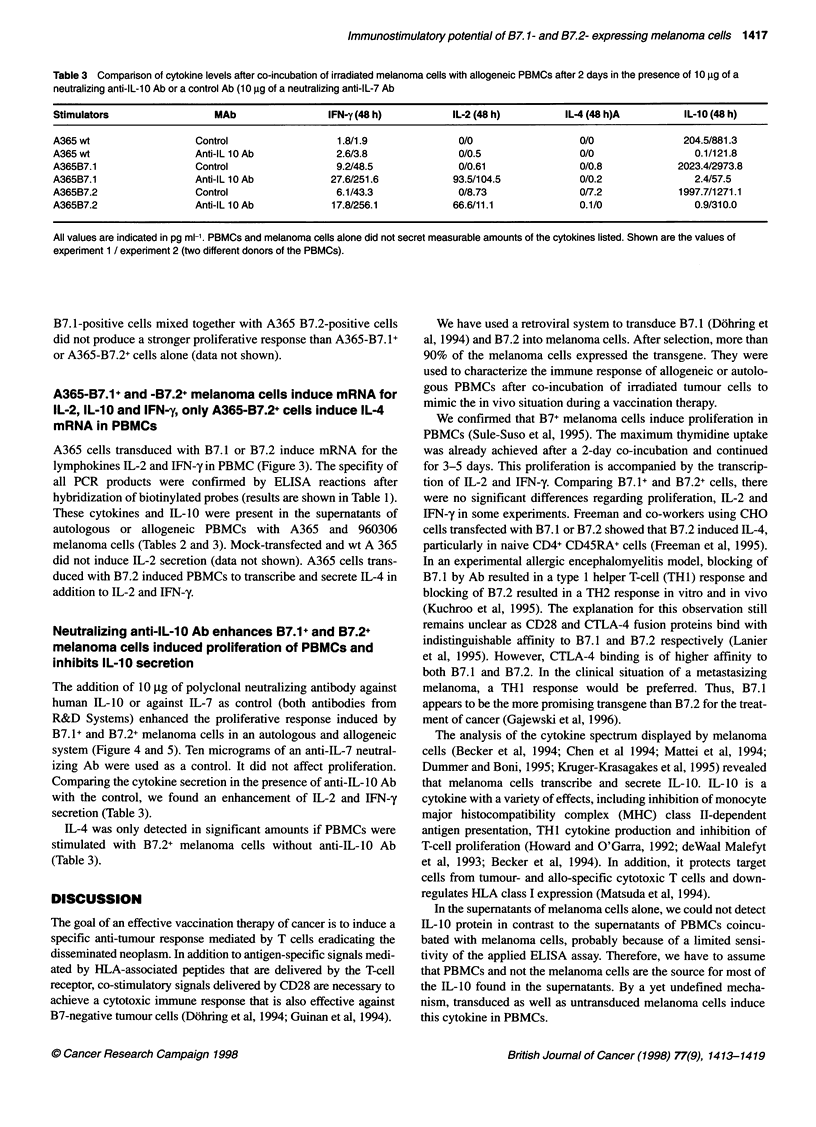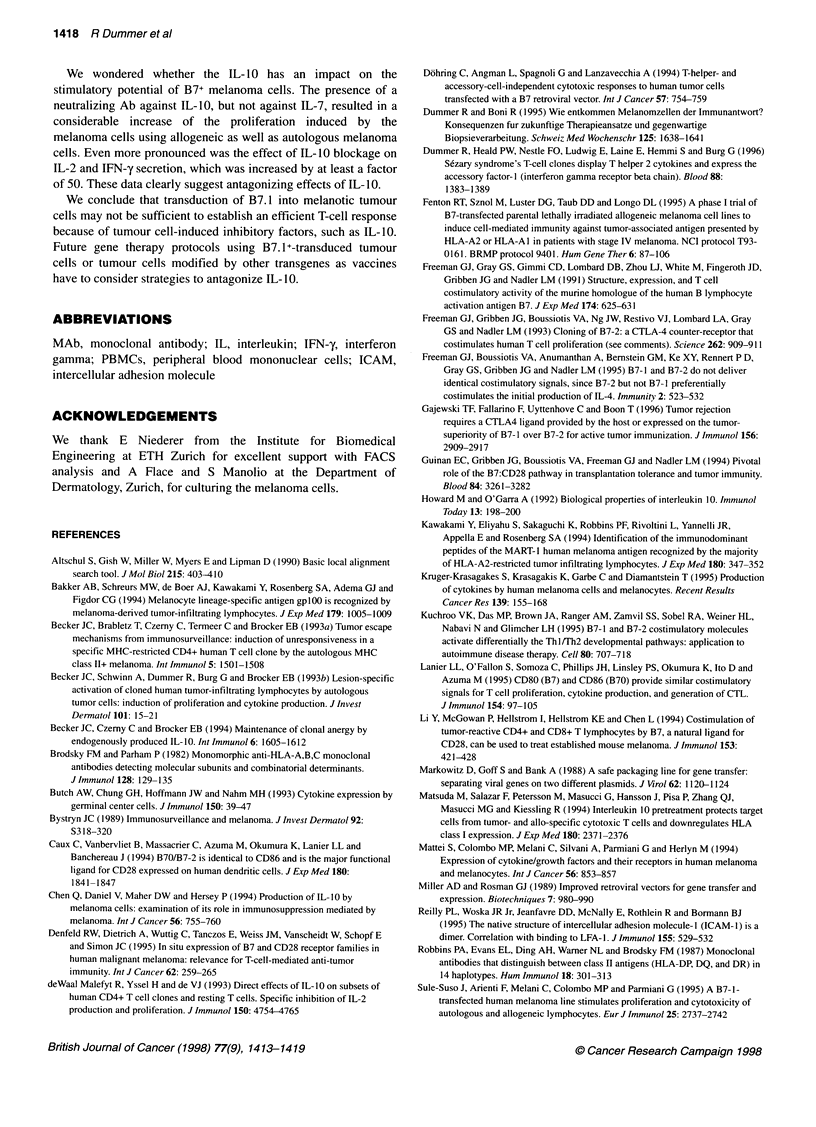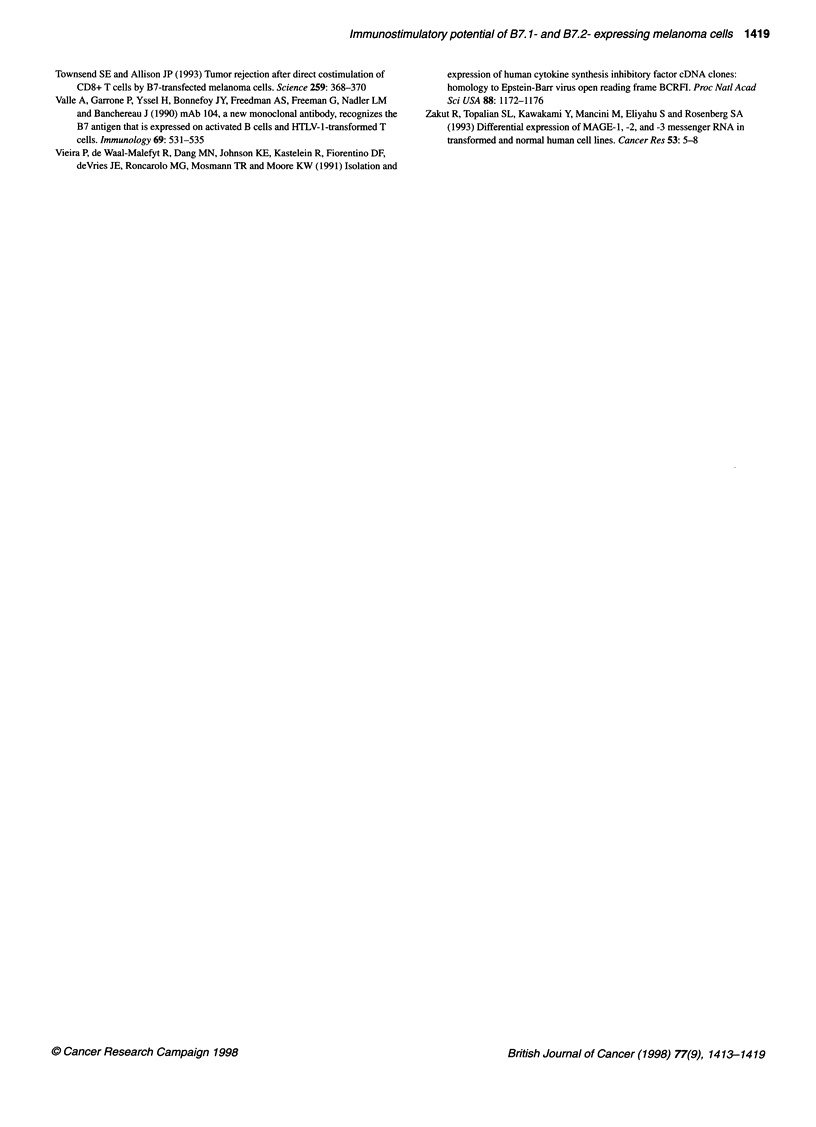# Immune stimulatory potential of B7.1 and B7.2 retrovirally transduced melanoma cells: suppression by interleukin 10.

**DOI:** 10.1038/bjc.1998.234

**Published:** 1998-05

**Authors:** R. Dummer, F. Y. Yue, J. Pavlovic, R. Geertsen, C. DÃ¶hring, K. Moelling, G. Burg

**Affiliations:** Department of Dermatology, University of Zurich Medical School, Switzerland.

## Abstract

**Images:**


British Journal of Cancer (1998) 77(9), 1413–1419
© 1998 Cancer Research Campaign

### Immune stimulatory potential of B7.1 and B7.2 retrovirally transduced melanoma cells: suppression by interleukin 10

**R Dummer[1], F-Y Yue[1], J Pavlovic[2], R Geertsen[1], C Döhring[3], K Moelling[2] and G Burg[1]**

[1]Department of Dermatology, University of Zurich Medical School, Gloriastrasse 31, CH-8091 Zurich, Switzerland; [2]Institute for Medical Virology, University of Zurich Medical School, Gloriastrasse 30, CH-8091 Zurich, Switzerland; [3]Basel Institute for Immunology, Grenzacher Strasse 487, CH-4005 Basle, Switzerland

**Summary** The immunostimulatory capacities of B7.1-and B7.2- expressing melanoma cells were investigated. A365, 960306 and 950504 melanomas, established from nodular melanoma lesions, were retrovirally transduced. Irradiated B7–, B7.1+ and B7.2+ melanoma cells were co-cultured with autologous or allogeneic peripheral blood mononuclear cells (PBMCs). Proliferation was assessed by [$^3$H]thymidine uptake. mRNA encoding for interleukin 2 (IL-2), IL-4, IL-10 and interferon gamma (IFN-γ) was determined. IFN-γ, IL-2, IL-4 and IL-10 secretion were quantitated by ELISA. B7.1+ and B7.2+ melanomas induced proliferation of PBMCs and mRNA for IL-2 and IFN-γ. After co-incubation of transduced melanoma cells with PBMCs, high levels of IL-10 were detectable in the supernatant. The presence of neutralizing anti-IL-10 antibodies resulted in enhanced proliferation and IL-2 and IFN-γ secretion. Our data indicate that B7.1- and B7.2-transduced melanoma cells trigger lymphocytic proliferation with transcription of IL-10, IL-2 and IFN-γ. Blocking of IL-10 augments these effects. Gene therapy protocols using tumour cells as a vaccine have to consider the adverse effects of IL-10.

**Keywords:** melanoma; gene therapy; retroviral gene transfer; B7.1 (CD80); B7.2 (CD86); transduction; T-cell response; interleukin 2; interleukin 4; interleukin 10; interferon gamma

Melanoma is an antigenic tumour (Bystryn, 1989). Several proteins have been identified that are presented as peptides in the grove of the HLA-I complex. These proteins include members of the MAGE family (Zakut et al, 1993), gp 100 (Bakker et al, 1994) or tyrosinase (Kawakami et al, 1994).

Although melanoma presents various specific antigens associated with HLA-I, it does not induce an immune response, which results in an elimination of the malignant cells. It is speculated that melanoma cells induce tolerance instead of activation by presenting antigens without the respective co-stimulatory signals (Becker et al, 1993*a*). These co-stimulatory signals can be provided by B7.1 (CD80) or B7.2 (CD86), two molecules capable of delivering co-stimulatory signals to T cells via CD 28 (Becker et al, 1993*a*; Freeman et al, 1991, 1993; Guinan et al, 1994).

Thus, the immunogenicity of tumour cells is increased if the cells are transfected with human B7.1 (Döhring et al, 1994). Expression of the co-stimulatory ligand B7 on melanoma cells has been shown to induce the rejection of a murine melanoma in vivo (Townsend and Allison, 1993). In addition, treatment of mice bearing an 8-day established melanoma by intraperitoneal injection of B7$^+$ tumour cells resulted in complete tumour regression and cure (Li et al, 1994). In human melanomas, B7.1 expression was found only in regressing lesions (Denfeld et al, 1995). Consequently, there are human gene therapy protocols for melanoma that apply melanoma cells genetically engineered to express B7.1 (Fenton et al, 1995).

*Received 2 January 1997*
*Revised 14 October 1997*
*Accepted 16 October 1997*

*Correspondence to:* R Dummer

We analysed the immunostimulatory potential of a B7.1- and B7.2-transduced human melanoma cell line. We showed that B7.1- and B7.2-transduced melanoma cells induced proliferation and expression of IL-2 and IFN-γ. In addition, we demonstrated that B7.2-transduced melanoma cells induced IL-4, and that the presence of IL-10 suppressed the response of PBMCs.

### MATERIALS AND METHODS

### Cell culture: melanoma cells and peripheral blood mononuclear cells (PBMCs)

Human PBMCs were obtained from healthy volunteers or melanoma patients after informed consent, and were separated by Ficoll-Hypaque gradient centrifugation. After separation, the cells were washed twice and immediately resuspended in complete medium (CM) consisting of RPMI 1640 (Gibco/BRL), Eggenstein, Germany) supplemented with 10% of fetal bovine serum (FBS) (Gibco/BRL), 2 mM L-glutamine (Seromed, Berlin, Germany), 10 mM sodium pyruvate (Seromed), 100 U ml$^{-1}$ penicillin (Seromed), 100 mg ml$^{-1}$ streptomycin (Seromed) and 50 mg of gentamycin (Seromed). Before proliferation experiments, lymphocytes were enriched by plastic adherence over night and collection of the non-adherent cells.

The melanoma cell line A365 (kindly provided by U Reinhold, Department of Dermatology, Bonn, Germany) was also cultured in CM. The 950504 melanoma cells were established from a primary nodular melanoma of a 27-year-old female patient, the 960306 from a skin metastasis of a 36-year-old man as described earlier (Becker et al, 1993*b*). Both melanomas expressed human HLA class I, as determined by flow cytometry with the MAb W6/32, and ICAM-1, as determined with the MAb 8.4A6, but they were negative for HLA-DR (data not shown).

**Table 1** Allogeneic PBMCs were stimulated with A365 melanoma cells that were either untransfected (wt) or transfected with B7.1+ or B7.2. After 2 days of incubation, mRNA was extracted, reverse transcribed and the cDNA amplified with specific primers. PCR products were detected by PCR-ELISA

| | OD ± s.d. (405–492 nm) | | | | |
|---|---|---|---|---|---|
| **Samples** | **IL-2** | **IL-4** | **IL-10** | **IFN-γ** | **β-Actin** |
| A365wt | 0.083 ± 0.007 | 0.094 ± 0.008 | 2.497 ± 0.192 | 0.048 ± 0.003 | 0.932 ± 0.078 |
| A365B7.1 | 0.948 ± 0.087 | 0.105 ± 0.012 | 2.463 ± 0.213 | 0.434 ± 0.031 | 0.909 ± 0.084 |
| A365B7.2 | 0.834 ± 0.076 | 0.394 ± 0.024 | 1.055 ± 0.147 | 0.297 ± 0.019 | 1.001 ± 0.109 |
| Positive control | 1.001 ± 0.098 | 1.028 ± 0.094 | 1.618 ± 0.151 | 1.006 ± 0.021 | 1.008 ± 0.099 |
| Negative control | 0.068 ± 0.005 | 0.055 ± 0.004 | 0.074 ± 0.006 | 0.053 ± 0.003 | 0.056 ± 0.004 |

**Figure 1** A365 melanoma cells transduced with viral supernatants containing human B7.1 or B7.2 cDNA were stained with anti-CD80-FITC MAb, with anti-CD86-FITC MAb or with saturating concentrations of anti-human CTLA4-IgG fusion protein, followed by FITC-conjugated goat anti-human IgG Ab. Samples were analysed by flow cytometry, the histograms show log fluorescence vs number of cells

### Construction of B7.1 and B7.2 retroviral vectors

The B7.1 and B7.2 open reading frames were amplified from Epstein–Barr virus B-cell cDNA (Döhring et al, 1994). Amplification was carried out on a DNA Thermal Cycler (Perkin-Elmer Cetus) using the following conditions: 94°C, 1 min, then 25 cycles of 94°C, 15 s, 60°C, 30 s, 72°C, 45 s, followed by 72°C, 10 min. The amplified PCR product was cloned into the retroviral vector pLXSN (Miller and Rosman, 1989). The sequence of the B7 inserts was verified to be identical to the published sequence (Freeman et al, 1991, 1993) (data not shown). Bacteria were transformed by electroporation, selected on ampicillin-containing agar plates, and plasmid DNA from resistant colonies was purified. The retroviral constructs were packaged in an ecotropic packaging system using the GP+E cell line. Amphotropic packaging was performed using the cell line GP+*env*AM12 (Markowitz et al, 1988). As determined by infection on NIH3T3 cell layers, the titre of the viral supernatants was in excess of $10^6$ ml$^{-1}$. These supernatants were used to transduce the A365 and 950504 melanoma cells with B7.1 or B7.2. After 2 days, 1 mg ml$^{-1}$ G418 (Gibco/BRL) was added to the culture for the selection of resistant cells (Döhring et al, 1994).

### Proliferation assays

B7.1+, B7.2+, mock-transfected and B7− A365 melanoma cells were tested for their capacity to stimulate allogeneic T cells. As there was no difference between mock-transfected and wt A365

© Cancer Research Campaign 1998

**Figure 2** Untransduced A365, A365-B7.1+ or A365-B7.2+ cells (2 × 10[4] cells per well irradiated for 60 s) incubated with resting allogenic PBMCs for 0.5, 2, 4, 6, 8, 10 days. Thereafter, the cultures were labelled for 18 h with [$^3$H]thymidine, and the radioactivity incorporated was assessed by liquid scintillation counting (c.p.m. mean ± s.e.m., $n$ = 3, s.e.m. < 200 c.p.m. not shown). A good discrimination between transduced and wt cell stimulation was already possible after 2 days

**Figure 3** RT-PCR for IL-2 and IFN-γ in allogenic PBMCs stimulated by B7- A365, A365-B7.1+ or A365-B7.2+ cells. The beta-actin control extinction after hybridization is shown in Table 1

**Table 2** Cytokine secretion of autologous and allogeneic PBMCs stimulated with B7-, B7.1+ or B7.2+ transduced melanoma cells

| Cells | IL-2 | IL-4 | IL-10 | IFN-γ |
|---|---|---|---|---|
| *Autologous* | | | | |
| M960306wt | 0 | 0 | 355.0 | 0 |
| M960306B7.1 | 3.9 | 0.6 | 167.1 | 10.1 |
| M960306B7.2 | 8.8 | 2.1 | 109.1 | 22.0 |
| *Allogeneic* | | | | |
| M960306wt | 0 | 0 | 270.0 | 0 |
| M960306B7.1 | 8.5 | 0 | 278.0 | 84.0 |
| M960306B7.2 | 3.5 | 0.5 | 280.0 | 13.4 |

(data not shown), only wt cells were used as control. PBMCs were cultured with irradiated stimulator cells in 96-well flat-bottomed microtitre plates in 200 µl of RPMI-5% human serum. In some experiments, PBMCs were cultured with 10[4] irradiated tumour cells in U-bottomed microtitre plates. After 2–3 days (or later if indicated), the cultures were labelled with [$^3$H]thymidine, and the radioactivity incorporated was assessed by liquid scintillation counting. The identical approach was used to analyse the proliferation induced by untransduced and B7.1+ 950504 melanoma cells co-cultured with autologous PBMCs.

For mRNA extraction 3 × 10[6] PBMCs were coincubated with 3 × 10[5] irradiated melanoma cells in six-well flat-bottomed plates for 2 days. Supernatants for cytokine measurements and PBMCs for mRNA extraction were harvested on day 2.

### Flow cytometric analysis

The following primary MAbs were used at the concentrations indicated: W6.32 (supernatant purified on protein G sepharose; mouse IgG2a) reacting with a monomorphic determinant on the human HLA class I A, B, C molecules (Brodsky and Parham, 1982; 3.6 µg ml$^{-1}$); L243 (Becton Dickinson, San Jose, CA, USA; mouse IgG2a) recognizing human HLA class II DR epitopes (Robbins et al, 1987; 2.5 µg ml$^{-1}$); 8.4A6 (Ancell, Bayport, MN, USA; mouse IgG1) recognizing D2 domain of the human CD54 (ICAM-1) molecule (Reilly et al, 1995; 5.0 µg ml$^{-1}$). MAB104 (Immunotech, Marseille, France; mouse IgG1) reacting with B7.1 co-stimulatory molecule (Valle et al, 1990; 20 µg ml$^{-1}$); and BU63 (Ancell; mouse IgG1) reacting with B7.2 co-stimulatory molecule (Caux et al, 1994; 4.2 µg ml$^{-1}$). The hCTLA4-IgG fusion protein, consisting of human CTLA4 extracellular domain and human IgG1 constant domains, binds to both B7.1 and B7.2 molecules with even higher affinity than CD28, the natural ligand for these receptors (supernatant purified on protein G sepharose; Döhring et al, 1994; 2.0 µg ml$^{-1}$).

Adherent cells were detached with a solution containing 0.05% trypsin and 0.02% EDTA (Seromed), inactivated with fresh media and washed once with F-PBS [phosphate-buffered saline without $Ca^{++}/Mg^{++}$ containing 1% fetal calf serum (FCS)] and 0.2 mg ml$^{-1}$ sodium azide. Approximately 5–10 × 10[5] cells each were incubated with different MAbs at concentrations indicated above in a total volume of 200 µl for 45 min on ice in the dark. After washing, non-conjugated antibodies were stained with 1:30 dilution (in F-PBS) of either FITC-conjugated rabbit anti-mouse or anti-human IgG secondary antibody (Dako, Glostrup, Denmark) for 30 min on ice in the dark. In all cases, cells were also stained with the appropriate isotype-matched control antibodies, non-specific mouse IgG1 or IgG2a (Becton Dickinson). After washing and fixation with 0.5% formaldehyde in F-PBS, fluorescence of the cells was measured by an Epics Profile II cytofluorometer (Coulter, Miami, FL, USA). Non-viable cells were gated out. FACS data are presented as log of fluorescence intensity vs counts.

### Reverse transcriptase–polymerase chain reaction (RT-PCR)

RNA extraction from PBMCs or melanoma cells was performed as follows: the cell pellet was taken up in buffer A (10 mM Hepes, 10 mM potassium chloride, 1 mM EDTA, 1 mM EGTA) and lysed by vortexing with the addition of 1:16 vol. of 10% NP40. The supernatant was added to an equal volume of buffer B (7 M urea, 1% sodium dodecyl sulphate, 0.35 M sodium chloride). After one phenol–chloroform extraction and one chloroform extraction the RNA was precipitated with ethanol–glycogen and dissolved in DEPC-water. The RNA was quantified by reading absorbance at 260 nm ($A_{260}$); 2–4 µg of RNA were used to synthesize cDNA (M-MuLV reverse transcriptase from New England Biolabs).

### PCR analysis

PCR was performed with the incubation buffer supplied with the *Taq* DNA polymerase (Boehringer, Mannheim, Germany), with

© Cancer Research Campaign 1998 

PCR DIG labelling nucleotide mix (Boehringer) and with 2.0 μM oligonucleotide primers. All cDNAs were first amplified with primers for beta-actin to test the quality and quantity of the cDNA. Only satisfactory cDNA was then subjected to PCR with primers for IL-2, IL-4, IL-10 and IFN-γ. Primers were selected using the Oligo Primer Analysis Software program version 4.0 (National Biosciences), except for primers for IL-10, which have published sequences: upper primer pos. 323, 27 nt (Vieira et al, 1991); lower primer pos. 646, 27 nt (Butch et al, 1993). Primer sequences were sent to genebank to exclude cross-binding to other published sequences (Altschul et al; 1990) and were published recently (Dummer et al, 1996). PCR was performed with Perkin Elmer 9600 GenAmp cyclers. Annealing temperatures were 55°C for IFN-γ, IL-2 and IL-12p35; 62°C for IL-4, IL-5, IL-7 and IL12p40; and 66°C for IL-10 and IL-13. One cycle (93°C, 2 min 30 s, annealing temperature 1 min 30 s, 72°C, 1 min 30 s) was followed by 30 cycles (94°C, 30 s, annealing temperature, 30 s, 72°C, 1 min 30 s). An aliquot of PCR product was electrophoresed on a 1.6% agarose gel and visualized by ethidium bromide staining. In all PCR reactions, positive controls from various cell lines (see Table 1) and water as negative control were included.

### PCR ELISA

For the PCR enzyme-linked immunosorbent assay (ELISA), nucleotide probes were selected with the Oligo software for the sequences of the cDNA fragment amplified (see Table 1) by the specific primers and obtained biotinylated (Microsynth, Balgach, Switzerland). The specifity of all PCR products was confirmed with this method. PCR ELISA was performed according to kit directions (Boehringer). Hybridization was specific at 45°C for all probes except for IL-10. To exclude non-specific binding, hybridization with the IL-10 probes was performed at 50°C. The specific capture probe/PCR product hybrids were bound to streptavidin-coated microtitre plates via the biotin label of the probes. After washing, the immobilized hybrids were treated with anti-DIG peroxidase-conjugated antibody and ABTS, a substrate for the peroxidase. The plates were measured by reading $A_{492}$ and values that were higher than 2× the reading of the PCR reaction mix with water instead of cDNA were judged to be positive for the expression of the cytokine in question (Dummer et al, 1996).

**Figure 4** Melanoma-induced proliferation of PBMCs in an allogeneic system after neutralization of IL-10 by an anti-IL-10 Ab. Neutralization of IL-10 results in a doubling of the proliferation (c.p.m. mean ± s.e.m., $n = 3$)

### Cytokine ELISA

Using commercially available ELISAs, the supernatants of PBMCs were screened for the cytokines IFN-γ, IL-2, IL-4, IL-10; after co-incubation with melanoma cells (ELISAs obtained from R&D Systems, Minneapolis, MN, USA).

### RESULTS

### Transduced melanoma cells express B7.1 or B7.2

Human A365 cells were stably transduced with viral supernatants containing human B7.1 or human B7.2 cDNA. 950504 melanoma cells were transduced with B7.1 only. Transduced cells were strongly stained with anti-CD80-FITC MAb, anti-CD86-FITC MAb or with saturating amounts of human CTLA4-IgG fusion protein, followed by FITC-conjugated goat anti-human IgG. Flow cytometric analysis revealed high levels of human CTLA4-IgG binding to both B7.1- and B7.2-transduced A365 or 950504 melanoma cells. Figure 1 shows the cytometric analysis of B7.1- and B7.2-positive A365 and A365 wt.

### A365 and 950504 melanoma cells express IL-10

Using the RT-PCR ELISA technique, we detected mRNA encoding for IL-10 in A365 and 950504 melanoma cells (Dummer and Boni, 1995). A365 cells were also positive for IL-5 and IL-7, but not for IL-2, IL-4, IL-12p35/40, IL-13 or IFN-γ, as determined by RT-PCR ELISA (data not shown). The presence of anti-IL-10 Ab did not affect the expression of B7.1/B7.2 molecules on the surface of melanoma cells (data not shown).

### B7.1$^+$ and B7.2$^+$ melanoma cells induce proliferation of PBMCs

After irradiation, the B7.1- and B7.2-transduced cells induced a significantly higher proliferative response than untransduced cells or A365 cells transduced with empty vector, when autologous or allogeneic PBMCs were used as responders; see Figure 2. A365

**Figure 5** Determination of melanoma-induced proliferation of PBMCs in an autologous system after neutralization of IL-10 by an anti-IL-10 Ab. Neutralization of IL-10 results in a doubling of the proliferation (c.p.m. mean ± s.e.m., $n = 3$)

© Cancer Research Campaign 1998

**Table 3** Comparison of cytokine levels after co-incubation of irradiated melanoma cells with allogeneic PBMCs after 2 days in the presence of 10 μg of a neutralizing anti-IL-10 Ab or a control Ab (10 μg of a neutralizing anti-IL-7 Ab

| Stimulators | MAb | IFN-γ (48 h) | IL-2 (48 h) | IL-4 (48 h)A | IL-10 (48 h) |
|---|---|---|---|---|---|
| A365 wt | Control | 1.8/1.9 | 0/0 | 0/0 | 204.5/881.3 |
| A365 wt | Anti-IL 10 Ab | 2.6/3.8 | 0/0.5 | 0/0 | 0.1/121.8 |
| A365B7.1 | Control | 9.2/48.5 | 0/0.61 | 0/0.8 | 2023.4/2973.8 |
| A365B7.1 | Anti-IL 10 Ab | 27.6/251.6 | 93.5/104.5 | 0/0.2 | 2.4/57.5 |
| A365B7.2 | Control | 6.1/43.3 | 0/8.73 | 0/7.2 | 1997.7/1271.1 |
| A365B7.2 | Anti-IL 10 Ab | 17.8/256.1 | 66.6/11.1 | 0.1/0 | 0.9/310.0 |

All values are indicated in pg $ml^{-1}$. PBMCs and melanoma cells alone did not secret measurable amounts of the cytokines listed. Shown are the values of experiment 1 / experiment 2 (two different donors of the PBMCs).

B7.1-positive cells mixed together with A365 B7.2-positive cells did not produce a stronger proliferative response than A365-B7.1$^+$ or A365-B7.2$^+$ cells alone (data not shown).

### A365-B7.1$^+$ and -B7.2$^+$ melanoma cells induce mRNA for IL-2, IL-10 and IFN-γ, only A365-B7.2$^+$ cells induce IL-4 mRNA in PBMCs

A365 cells transduced with B7.1 or B7.2 induce mRNA for the lymphokines IL-2 and IFN-γ in PBMC (Figure 3). The specifity of all PCR products were confirmed by ELISA reactions after hybridization of biotinylated probes (results are shown in Table 1). These cytokines and IL-10 were present in the supernatants of autologous or allogeneic PBMCs with A365 and 960306 melanoma cells (Tables 2 and 3). Mock-transfected and wt A 365 did not induce IL-2 secretion (data not shown). A365 cells transduced with B7.2 induced PBMCs to transcribe and secrete IL-4 in addition to IL-2 and IFN-γ.

### Neutralizing anti-IL-10 Ab enhances B7.1$^+$ and B7.2$^+$ melanoma cells induced proliferation of PBMCs and inhibits IL-10 secretion

The addition of 10 μg of polyclonal neutralizing antibody against human IL-10 or against IL-7 as control (both antibodies from R&D Systems) enhanced the proliferative response induced by B7.1$^+$ and B7.2$^+$ melanoma cells in an autologous and allogeneic system (Figure 4 and 5). Ten micrograms of an anti-IL-7 neutralizing Ab were used as a control. It did not affect proliferation. Comparing the cytokine secretion in the presence of anti-IL-10 Ab with the control, we found an enhancement of IL-2 and IFN-γ secretion (Table 3).

IL-4 was only detected in significant amounts if PBMCs were stimulated with B7.2$^+$ melanoma cells without anti-IL-10 Ab (Table 3).

### DISCUSSION

The goal of an effective vaccination therapy of cancer is to induce a specific anti-tumour response mediated by T cells eradicating the disseminated neoplasm. In addition to antigen-specific signals mediated by HLA-associated peptides that are delivered by the T-cell receptor, co-stimulatory signals delivered by CD28 are necessary to achieve a cytotoxic immune response that is also effective against B7-negative tumour cells (Döhring et al, 1994; Guinan et al, 1994).

We have used a retroviral system to transduce B7.1 (Döhring et al, 1994) and B7.2 into melanoma cells. After selection, more than 90% of the melanoma cells expressed the transgene. They were used to characterize the immune response of allogeneic or autologous PBMCs after co-incubation of irradiated tumour cells to mimic the in vivo situation during a vaccination therapy.

We confirmed that B7$^+$ melanoma cells induce proliferation in PBMCs (Sule-Suso et al, 1995). The maximum thymidine uptake was already achieved after a 2-day co-incubation and continued for 3–5 days. This proliferation is accompanied by the transcription of IL-2 and IFN-γ. Comparing B7.1$^+$ and B7.2$^+$ cells, there were no significant differences regarding proliferation, IL-2 and IFN-γ in some experiments. Freeman and co-workers using CHO cells transfected with B7.1 or B7.2 showed that B7.2 induced IL-4, particularly in naive CD4$^+$ CD45RA$^+$ cells (Freeman et al, 1995). In an experimental allergic encephalomyelitis model, blocking of B7.1 by Ab resulted in a type 1 helper T-cell (TH1) response and blocking of B7.2 resulted in a TH2 response in vitro and in vivo (Kuchroo et al, 1995). The explanation for this observation still remains unclear as CD28 and CTLA-4 fusion proteins bind with indistinguishable affinity to B7.1 and B7.2 respectively (Lanier et al, 1995). However, CTLA-4 binding is of higher affinity to both B7.1 and B7.2. In the clinical situation of a metastasizing melanoma, a TH1 response would be preferred. Thus, B7.1 appears to be the more promising transgene than B7.2 for the treatment of cancer (Gajewski et al, 1996).

The analysis of the cytokine spectrum displayed by melanoma cells (Becker et al, 1994; Chen et al 1994; Mattei et al, 1994; Dummer and Boni, 1995; Kruger-Krasagakes et al, 1995) revealed that melanoma cells transcribe and secrete IL-10. IL-10 is a cytokine with a variety of effects, including inhibition of monocyte major histocompatibility complex (MHC) class II-dependent antigen presentation, TH1 cytokine production and inhibition of T-cell proliferation (Howard and O'Garra, 1992; deWaal Malefyt et al, 1993; Becker et al, 1994). In addition, it protects target cells from tumour- and allo-specific cytotoxic T cells and down-regulates HLA class I expression (Matsuda et al, 1994).

In the supernatants of melanoma cells alone, we could not detect IL-10 protein in contrast to the supernatants of PBMCs coincubated with melanoma cells, probably because of a limited sensitivity of the applied ELISA assay. Therefore, we have to assume that PBMCs and not the melanoma cells are the source for most of the IL-10 found in the supernatants. By a yet undefined mechanism, transduced as well as untransduced melanoma cells induce this cytokine in PBMCs.

© Cancer Research Campaign 1998 

We wondered whether the IL-10 has an impact on the stimulatory potential of B7+ melanoma cells. The presence of a neutralizing Ab against IL-10, but not against IL-7, resulted in a considerable increase of the proliferation induced by the melanoma cells using allogeneic as well as autologous melanoma cells. Even more pronounced was the effect of IL-10 blockage on IL-2 and IFN-γ secretion, which was increased by at least a factor of 50. These data clearly suggest antagonizing effects of IL-10.

We conclude that transduction of B7.1 into melanotic tumour cells may not be sufficient to establish an efficient T-cell response because of tumour cell-induced inhibitory factors, such as IL-10. Future gene therapy protocols using B7.1+-transduced tumour cells or tumour cells modified by other transgenes as vaccines have to consider strategies to antagonize IL-10.

### ABBREVIATIONS

MAb, monoclonal antibody; IL, interleukin; IFN-γ, interferon gamma; PBMCs, peripheral blood mononuclear cells; ICAM, intercellular adhesion molecule

### ACKNOWLEDGEMENTS

We thank E Niederer from the Institute for Biomedical Engineering at ETH Zurich for excellent support with FACS analysis and A Flace and S Manolio at the Department of Dermatology, Zurich, for culturing the melanoma cells.

### REFERENCES

Altschul S, Gish W, Miller W, Myers E and Lipman D (1990) Basic local alignment search tool. *J Mol Biol* **215**: 403–410

Bakker AB, Schreurs MW, de Boer AJ, Kawakami Y, Rosenberg SA, Adema GJ and Figdor CG (1994) Melanocyte lineage-specific antigen gp100 is recognized by melanoma-derived tumor-infiltrating lymphocytes. *J Exp Med* **179**: 1005–1009

Becker JC, Brabletz T, Czerny C, Termeer C and Brocker EB (1993*a*) Tumor escape mechanisms from immunosurveillance: induction of unresponsiveness in a specific MHC-restricted CD4+ human T cell clone by the autologous MHC class II+ melanoma. *Int Immunol* **5**: 1501–1508

Becker JC, Schwinn A, Dummer R, Burg G and Brocker EB (1993*b*) Lesion-specific activation of cloned human tumor-infiltrating lymphocytes by autologous tumor cells: induction of proliferation and cytokine production. *J Invest Dermatol* **101**: 15–21

Becker JC, Czerny C and Brocker EB (1994) Maintenance of clonal anergy by endogenously produced IL-10. *Int Immunol* **6**: 1605–1612

Brodsky FM and Parham P (1982) Monomorphic anti-HLA-A,B,C monoclonal antibodies detecting molecular subunits and combinatorial determinants. *J Immunol* **128**: 129–135

Butch AW, Chung GH, Hoffmann JW and Nahm MH (1993) Cytokine expression by germinal center cells. *J Immunol* **150**: 39–47

Bystryn JC (1989) Immunosurveillance and melanoma. *J Invest Dermatol* **92**: S318–320

Caux C, Vanbervliet B, Massacrier C, Azuma M, Okumura K, Lanier LL and Banchereau J (1994) B70/B7-2 is identical to CD86 and is the major functional ligand for CD28 expressed on human dendritic cells. *J Exp Med* **180**: 1841–1847

Chen Q, Daniel V, Maher DW and Hersey P (1994) Production of IL-10 by melanoma cells: examination of its role in immunosuppression mediated by melanoma. *Int J Cancer* **56**: 755–760

Denfeld RW, Dietrich A, Wuttig C, Tanczos E, Weiss JM, Vanscheidt W, Schopf E and Simon JC (1995) In situ expression of B7 and CD28 receptor families in human malignant melanoma: relevance for T-cell-mediated anti-tumor immunity. *Int J Cancer* **62**: 259–265

deWaal Malefyt R, Yssel H and de VJ (1993) Direct effects of IL-10 on subsets of human CD4+ T cell clones and resting T cells. Specific inhibition of IL-2 production and proliferation. *J Immunol* **150**: 4754–4765

Döhring C, Angman L, Spagnoli G and Lanzavecchia A (1994) T-helper- and accessory-cell-independent cytotoxic responses to human tumor cells transfected with a B7 retroviral vector. *Int J Cancer* **57**: 754–759

Dummer R and Boni R (1995) Wie entkommen Melanomzellen der Immunantwort? Konsequenzen fur zukunftige Therapieansatze und gegenwartige Biopsieverarbeitung. *Schweiz Med Wochenschr* **125**: 1638–1641

Dummer R, Heald PW, Nestle FO, Ludwig E, Laine E, Hemmi S and Burg G (1996) Sézary syndrome's T-cell clones display T helper 2 cytokines and express the accessory factor-1 (interferon gamma receptor beta chain). *Blood* **88**: 1383–1389

Fenton RT, Sznol M, Luster DG, Taub DD and Longo DL (1995) A phase I trial of B7-transfected parental lethally irradiated allogeneic melanoma cell lines to induce cell-mediated immunity against tumor-associated antigen presented by HLA-A2 or HLA-A1 in patients with stage IV melanoma. NCI protocol T93-0161. BRMP protocol 9401. *Hum Gene Ther* **6**: 87–106

Freeman GJ, Gray GS, Gimmi CD, Lombard DB, Zhou LJ, White M, Fingeroth JD, Gribben JG and Nadler LM (1991) Structure, expression, and T cell costimulatory activity of the murine homologue of the human B lymphocyte activation antigen B7. *J Exp Med* **174**: 625–631

Freeman GJ, Gribben JG, Boussiotis VA, Ng JW, Restivo VJ, Lombard LA, Gray GS and Nadler LM (1993) Cloning of B7-2: a CTLA-4 counter-receptor that costimulates human T cell proliferation (see comments). *Science* **262**: 909–911

Freeman GJ, Boussiotis VA, Anumanthan A, Bernstein GM, Ke XY, Rennert P D, Gray GS, Gribben JG and Nadler LM (1995) B7-1 and B7-2 do not deliver identical costimulatory signals, since B7-2 but not B7-1 preferentially costimulates the initial production of IL-4. *Immunity* **2**: 523–532

Gajewski TF, Fallarino F, Uyttenhove C and Boon T (1996) Tumor rejection requires a CTLA4 ligand provided by the host or expressed on the tumor-superiority of B7-1 over B7-2 for active tumor immunization. *J Immunol* **156**: 2909–2917

Guinan EC, Gribben JG, Boussiotis VA, Freeman GJ and Nadler LM (1994) Pivotal role of the B7:CD28 pathway in transplantation tolerance and tumor immunity. *Blood* **84**: 3261–3282

Howard M and O'Garra A (1992) Biological properties of interleukin 10. *Immunol Today* **13**: 198–200

Kawakami Y, Eliyahu S, Sakaguchi K, Robbins PF, Rivoltini L, Yannelli JR, Appella E and Rosenberg SA (1994) Identification of the immunodominant peptides of the MART-1 human melanoma antigen recognized by the majority of HLA-A2-restricted tumor infiltrating lymphocytes. *J Exp Med* **180**: 347–352

Kruger-Krasagakes S, Krasagakis K, Garbe C and Diamantstein T (1995) Production of cytokines by human melanoma cells and melanocytes. *Recent Results Cancer Res* **139**: 155–168

Kuchroo VK, Das MP, Brown JA, Ranger AM, Zamvil SS, Sobel RA, Weiner HL, Nabavi N and Glimcher LH (1995) B7-1 and B7-2 costimulatory molecules activate differentially the Th1/Th2 developmental pathways: application to autoimmune disease therapy. *Cell* **80**: 707–718

Lanier LL, O'Fallon S, Somoza C, Phillips JH, Linsley PS, Okumura K, Ito D and Azuma M (1995) CD80 (B7) and CD86 (B70) provide similar costimulatory signals for T cell proliferation, cytokine production, and generation of CTL. *J Immunol* **154**: 97–105

Li Y, McGowan P, Hellstrom I, Hellstrom KE and Chen L (1994) Costimulation of tumor-reactive CD4+ and CD8+ T lymphocytes by B7, a natural ligand for CD28, can be used to treat established mouse melanoma. *J Immunol* **153**: 421–428

Markowitz D, Goff S and Bank A (1988) A safe packaging line for gene transfer: separating viral genes on two different plasmids. *J Virol* **62**: 1120–1124

Matsuda M, Salazar F, Petersson M, Masucci G, Hansson J, Pisa P, Zhang QJ, Masucci MG and Kiessling R (1994) Interleukin 10 pretreatment protects target cells from tumor- and allo-specific cytotoxic T cells and downregulates HLA class I expression. *J Exp Med* **180**: 2371–2376

Mattei S, Colombo MP, Melani C, Silvani A, Parmiani G and Herlyn M (1994) Expression of cytokine/growth factors and their receptors in human melanoma and melanocytes. *Int J Cancer* **56**: 853–857

Miller AD and Rosman GJ (1989) Improved retroviral vectors for gene transfer and expression. *Biotechniques* **7**: 980–990

Reilly PL, Woska JR Jr, Jeanfavre DD, McNally E, Rothlein R and Bormann BJ (1995) The native structure of intercellular adhesion molecule-1 (ICAM-1) is a dimer. Correlation with binding to LFA-1. *J Immunol* **155**: 529–532

Robbins PA, Evans EL, Ding AH, Warner NL and Brodsky FM (1987) Monoclonal antibodies that distinguish between class II antigens (HLA-DP, DQ, and DR) in 14 haplotypes. *Hum Immunol* **18**: 301–313

Sule-Suso J, Arienti F, Melani C, Colombo MP and Parmiani G (1995) A B7-1-transfected human melanoma line stimulates proliferation and cytotoxicity of autologous and allogeneic lymphocytes. *Eur J Immunol* **25**: 2737–2742

© Cancer Research Campaign 1998

Townsend SE and Allison JP (1993) Tumor rejection after direct costimulation of CD8+ T cells by B7-transfected melanoma cells. *Science* **259**: 368–370

Valle A, Garrone P, Yssel H, Bonnefoy JY, Freedman AS, Freeman G, Nadler LM and Banchereau J (1990) mAb 104, a new monoclonal antibody, recognizes the B7 antigen that is expressed on activated B cells and HTLV-1-transformed T cells. *Immunology* **69**: 531–535

Vieira P, de Waal-Malefyt R, Dang MN, Johnson KE, Kastelein R, Fiorentino DF, deVries JE, Roncarolo MG, Mosmann TR and Moore KW (1991) Isolation and expression of human cytokine synthesis inhibitory factor cDNA clones: homology to Epstein-Barr virus open reading frame BCRFI. *Proc Natl Acad Sci USA* **88**: 1172–1176

Zakut R, Topalian SL, Kawakami Y, Mancini M, Eliyahu S and Rosenberg SA (1993) Differential expression of MAGE-1, -2, and -3 messenger RNA in transformed and normal human cell lines. *Cancer Res* **53**: 5–8

© Cancer Research Campaign 1998